# Hepatitis C virus NS2 and NS3/4A proteins are potent inhibitors of host cell cytokine/chemokine gene expression

**DOI:** 10.1186/1743-422X-3-66

**Published:** 2006-09-01

**Authors:** Pasi Kaukinen, Maarit Sillanpää, Sergei Kotenko, Rongtuan Lin, John Hiscott, Krister Melén, Ilkka Julkunen

**Affiliations:** 1 Department of Viral Diseases and Immunology, National Public Health Institute, Helsinki, Finland; 2 Department of Biochemistry and Molecular Biology, University of Medicine and Dentistry-New Jersey Medical School, Newark, NJ, USA; 3 Lady Davis Institute for Medical Research, Department of Microbiology & Immunology, McGill University, Montreal, Canada

## Abstract

**Background:**

Hepatitis C virus (HCV) encodes several proteins that interfere with the host cell antiviral response. Previously, the serine protease NS3/4A was shown to inhibit IFN-β gene expression by blocking dsRNA-activated retinoic acid-inducible gene I (RIG-I) and Toll-like receptor 3 (TLR3)-mediated signaling pathways.

**Results:**

In the present work, we systematically studied the effect of all HCV proteins on IFN gene expression. NS2 and NS3/4A inhibited IFN gene activation. NS3/4A inhibited the Sendai virus-induced expression of multiple IFN (IFN-α, IFN-β and IFN-λ1/IL-29) and chemokine (CCL5, CXCL8 and CXCL10) gene promoters. NS2 and NS3/4A, but not its proteolytically inactive form NS3/4A-S139A, were found to inhibit promoter activity induced by RIG-I or its adaptor protein Cardif (or IPS-1/MAVS/VISA). Both endogenous and transfected Cardif were proteolytically cleaved by NS3/4A but not by NS2 indicating different mechanisms of inhibition of host cell cytokine production by these HCV encoded proteases. Cardif also strongly colocalized with NS3/4A at the mitochondrial membrane, implicating the mitochondrial membrane as the site for proteolytic cleavage. In many experimental systems, IFN priming dramatically enhances RNA virus-induced IFN gene expression; pretreatment of HEK293 cells with IFN-α strongly enhanced RIG-I expression, but failed to protect Cardif from NS3/4A-mediated cleavage and failed to restore Sendai virus-induced IFN-β gene expression.

**Conclusion:**

HCV NS2 and NS3/4A proteins were identified as potent inhibitors of cytokine gene expression suggesting an important role for HCV proteases in counteracting host cell antiviral response.

## Background

Hepatitis C virus (HCV) (family *Flaviviridae*) is an enveloped virus with positive-sense, single-stranded RNA genome that causes both acute and persistent infections in humans associated with chronic hepatitis, cirrhosis and hepatocellular carcinoma. The HCV genome encodes for a polyprotein of about 3000 amino acids, which is cotranslationally and posttranslationally processed to mature proteins in the ER membrane. The core and envelope glycoproteins E1 and E2 form the structural proteins of the virion. Non-structural (NS) proteins NS2, NS3, NS4A, NS4B, NS5A and NS5B have important roles in the polyprotein processing and HCV replication [see for review [1]]. An alternative reading frame of the core region encodes for F protein, whose function is presently not known [2]. NS3 and NS4A proteins associate to form an active enzyme possessing RNA helicase and serine protease activities. NS3/4A has an ability to interfere with type I interferon (IFN) gene expression [3].

One of the host responses to virus infection is the production of chemokines and antiviral cytokines such as IFN-α and IFN-β. Virus-induced IFN production is also further enhanced by positive feedback mechanisms via type I IFNs [4]. The initial step for the induction of cytokine response in RNA virus infection is the activation of cellular dsRNA receptor systems, Toll-like receptor 3 (TLR3) [5] and DexH(D) RNA helicase, retinoic acid inducible gene-I (RIG-I) [6]. TLR3 and RIG-I act through adaptor proteins TRIF [7] and Cardif (also called as IPS-1/MAVS/VISA), respectively [8-11]. TRIF and Cardif mediate the activation of IκB kinase (IKK)α/β/γ complex and IKK-like kinases, IKKε and TBK1 [7-10,12], which leads to activation and nuclear translocation of NF-κB and IRF3 [13,14]. In the nucleus IRF3, NF-κB and AP-1 (ATF-2/c-Jun) transcription factors activate type I IFN and proinflammatory cytokine gene expression.

The first indication for the interferon antagonistic function of HCV NS3/4A was obtained in a study showing that NS3/4A inhibits IRF3 phosphorylation and activation [3]. Further studies demonstrated that NS3/4A disrupts both TLR3 and RIG-I-mediated signaling pathways [15-17]. TLR3 adaptor protein, TRIF, was found to be a direct proteolytic target of NS3/4A [18,19]. The RIG-I adaptor protein, Cardif, is another target for NS3/4A cleavage [11,20,21]. NS3/4A cleaves Cardif after Cys-508 residue, 32 amino acids from the C-terminus causing the release of Cardif from the mitochondrial outer membrane leading to its inability to function in RIG-I signaling [11,20].

Recent studies have mainly focused on the actions of NS3/4A in the IFN-β promoter regulation, while the role of other HCV proteins has remained less well characterized. We show here that NS3/4A blocks the gene expression of several chemokine and cytokine genes by degradating Cardif while NS2 protein inhibits gene expression (including IFN-β) with a different mechanism. Unlike in some other RNA virus infections, pretreatment of cells with IFN-α does not rescue virus-induced IFN gene expression, which is due to the lack of protection of Cardif from NS3/4A-mediated degradation. We also show that NS3/4A colocalizes with endogenous Cardif at the mitochondrial membrane suggesting that the mitochondrial membrane is the site of proteolytic cleavage of Cardif.

## Results

### HCV proteases NS2 and NS3/4A inhibit IFN-β promoter activity

**Figure 1:**
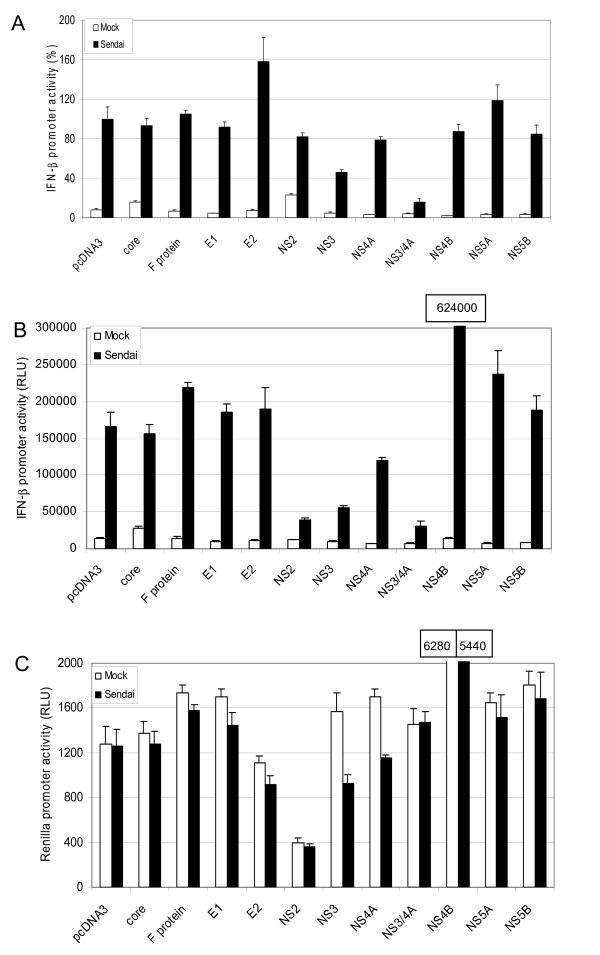
**HCV NS2 and NS3/4A inhibit IFN-β gene expression**. (A) The effect of expressed 11 HCV polypeptides on IFN-β promoter activity was studied in HEK293 cells by Luc reporter driven assay. The cells were transfected in triplicates with 1.0 μg HCV protein expression plasmids together with 0.1 μg firefly luciferase reporter under IFN-β promoter and 0.05 μg *Renilla *luciferase reporter (control) plasmids. Total DNA amount was balanced with the empty plasmid (pcDNA3.1(+)-FLAG). At 18 h after transfection the cells were infected with Sendai virus (MOI 5) or mock infected for 24 h, followed by collection of cells, preparation of cell lysates and measurement of luciferase activity. IFN-β promoter activities were normalized with *Renilla *luciferase activities. The activity of the sample that was transfected with empty pcDNA3 plasmids was assigned to 100%. Original values of IFN-β promoter (B) and *Renilla *luciferase (C) activities with HCV expression constructs are presented in the figures. Promoter activities were measured as triplicates and expressed as the means +/- standard deviations.

Recent studies have demonstrated that HCV NS3/4A protein complex interferes with IFN gene expression [3,15,19]. Since many other HCV proteins are also capable of interfering with host cell signalling pathways, we carried out a systematic analysis of all HCV proteins to determine their capacity to interfere with host cell signalling pathways regulating IFN gene expression. Expression plasmids encoding 11 HCV polypeptides were transfected into HEK293 cells together with IFN-β-Luc reporter plasmid; at 18 h after transfection, cells were infected with Sendai virus for 24 h, followed by preparation of cell lysates and measurement of luciferase activities (Fig. 1). Sendai virus was used since it is able to activate NF-κB, IRF and MAP kinase pathways that regulate the expression of chemokine and antiviral cytokine genes. HCV NS3 protein inhibited Sendai virus-induced IFN-β promoter activity approximately 50%, while the expression of NS3/4A complex reduced the promoter activity up to 85% (Fig. 1A). Strong inhibition by NS3/4A complex suggests that the association of NS4A cofactor with NS3 is crucial for the protein function. Viral envelope glycoprotein E2 was, in contrast, found to activate IFN-β promoter activity (ca. 60%) while other HCV proteins did not modulate the IFN-β promoter activity. This data indicates that serine protease NS3/4A is a specific inhibitor of IFN-β gene expression and other HCV proteins do not have similar function.

Original luciferase activity data, however, revealed that not only serine protease (NS3 and NS3/4A) but also HCV proteins NS2 and NS4B modulate IFN-β promoter activity (Fig. 1B). NS2 protein inhibited while NS4B protein activated the promoter 3–4-fold (Fig. 1B). Notably, NS2 protein also inhibited CCL5/RANTES and CXCL10/IP-10 promoters approx. 90% (data not shown). Both proteins (NS2 and NS4B) regulated TK promoter (*Renilla *luciferase) as well (Fig. 1C). *Renilla *luciferase activity was not affected by NS3/4A. The data suggests that NS2 protein, when expressed in high levels, is a general inhibitor of several cellular promoters. The significance of these observations requires further investigation (see Discussion).

### HCV NS3/4A inhibits several cytokine/chemokine promoters

**Figure 2:**
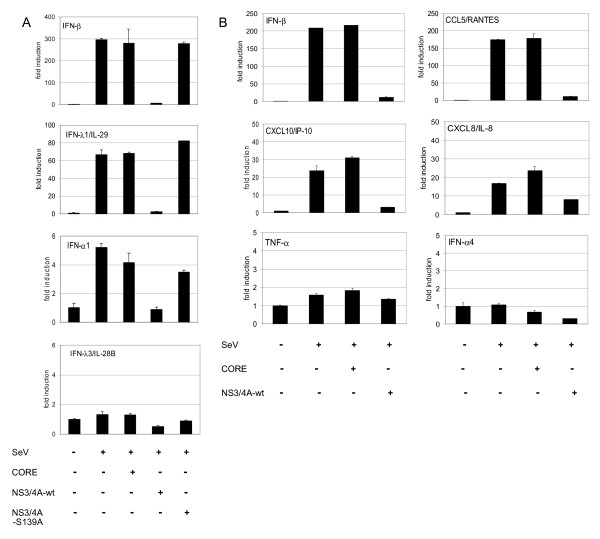
**NS3/4A protein is an effective antagonist for cytokine/chemokine promoters**. (A) IFN-β, IFN-λ1/IL-29, IFN-λ3/IL-28B and IFN-α1 gene promoter activities were studied in the presence of HCV core, NS3/4A-wt or NS3/4A-S139A after Sendai virus infection. (B) Cytokine/chemokine gene promoter activities were studied in the presence of HCV core or NS3/4A protein. The activities of IFN-β, CCL5/RANTES, CXCL8/IL-8, CXCL10/IP-10, TNF-α and IFN-α4 promoters in HCV core or NS3/4A-expressing HEK293 cells were measured after Sendai virus infection. HEK293 cells were treated as described in the legend for Figure 1. The activity of the sample that was transfected with empty pcDNA3 plasmids and mock infected was assigned to value of 1.

Previously, analysis of NS3/4A-mediated inhibition of IFN gene expression has been restricted to IFN-β gene. To further analyze whether the expression of other type I IFN or IFN-like genes is also inhibited we carried out transfection analyses with IFN-β, IFN-α1, IFN-λ1/IL-29 and IFN-λ3/IL-28B (almost identical to IFN-λ2 promoter) promoter-reporter contructs together with NS3/4A-wt and protease-inactive NS3/4A-S139A expression plasmids (Fig. 2A). HCV NS3/4A-wt efficiently inhibited Sendai virus-induced IFN-β, IFN-α1 and IFN-λ1/IL-29 promoter activities while the NS3/4A-S139A did not. Thus, IFN-α (α1), IFN-β and IFN-λ (λ1) genes are highly sensitive to the inhibitory effect of NS3/4A and the protease activity of NS3 is absolutely crucial for this inhibition.

The inhibitory effect of NS3/4A on other cytokine/chemokine gene promoters (IFN-β, CCL5/RANTES, CXCL10/IP-10, CXCL8/IL-8, TNF-α and IFN-α4) was next studied (Fig. 2B). NS3/4A, but not core protein, strongly inhibited Sendai virus-induced IFN-β, CCL5/RANTES and CXCL10/IP-10 promoters, while inhibition of CXCL8 promoter was more moderate being only ca. 50%. The promoters of IFN-λ3/IL-28B, TNF-α and IFN-α4 were practically not activated in Sendai virus-infected HEK293 cells suggesting that the transcriptional systems regulating these promoters are not effectively activated by Sendai virus or certain important components are missing in our model cell system. Altogether, our data suggest that NS3/4A protein is not only an effective antagonist of the IFN-β promoter but of other cytokine/chemokine promoters as well.

### Components of the RIG-I and TLR3/TLR4 pathway activate IFN-β promoter in HEK293 cells

**Figure 3:**
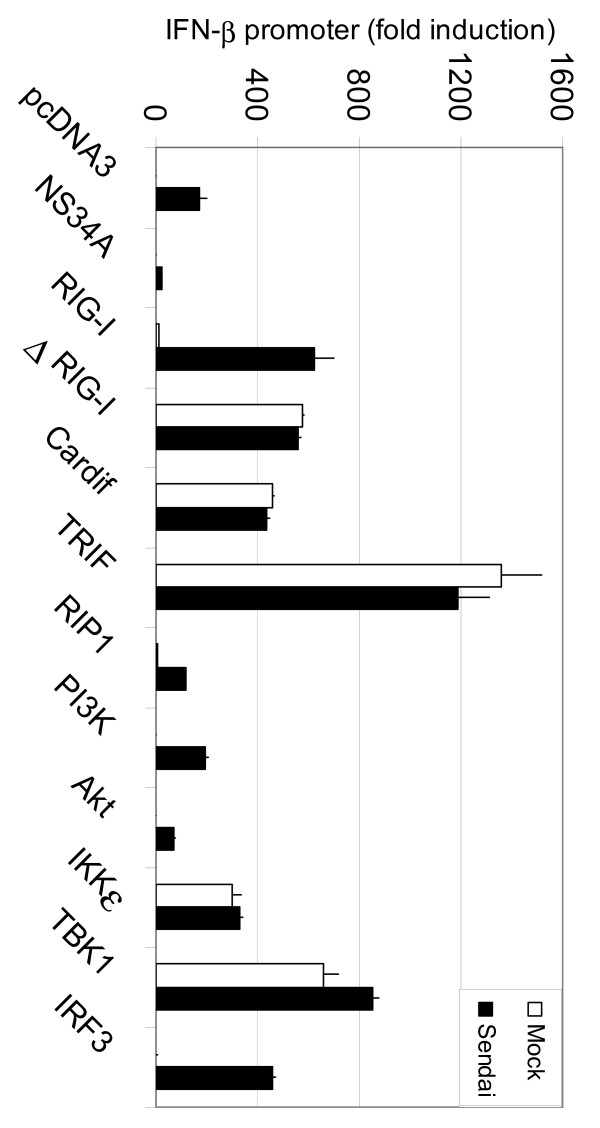
**Components of the RIG-I and TLR3/TLR4 pathway activate IFN-β promoter in HEK293 cells**. HEK293 cells were transfected with expression constructs (0.1 μg) for intracellular signaling molecules as shown in the figure and IFN-β reporter plasmid (0.1 μg). IFN-β promoter activities were measured in mock and Sendai virus-infected HEK293 cell lysates. The activity of the control sample (pcDNA3) was assigned to 1.

Recent studies have shown that many different signalling pathways, including RIG-I, TLR3, RIP1 or PI3K pathways are involved in IRF3 activation and IFN (IFN-β) gene expression [5,6,22,23]. We analyzed whether crucial components of these intracellular signal transduction pathways regulate IFN-β promoter activity in the presence or absence of activating virus infection. The data shows that constitutively active form of RIG-I (ΔRIG-I), Cardif, TRIF, IKKε and TBK1 directly activated IFN-β promoter (Fig. 3; white columns) and no further enhancement of the promoter activity was seen by Sendai virus infection (Fig. 3; black columns). The promoter activity was enhanced after Sendai virus infection in full-length RIG-I and IRF3-expressing cells suggesting that an additional signal through dsRNA is needed to activate the RIG-I pathway. It was recently shown that phosphoinositide 3-kinase (PI3K)-Akt pathway plays a role in TLR3-mediated IRF3 activation [23]. In our experiments, PI3K or Akt expression were not able to specifically induce IFN-β promoter activity suggesting that the expression of these molecules by themselves cannot induce IRF3 and IFN-β promoter activation. One may speculate that TBK1-mediated phosphorylation is crucial for initial IRF3 activation and the second phosphorylation step induced by PI3K pathway is needed for full transcriptional activity [23]. TRIF-associated RIP1 kinase was also not able to induce IFN-β promoter activity. Since RIP1 mediates NF-κB activation, RIP1 alone may not be sufficient to activate IFN gene expression [22]. Our data are in line with other reports showing that RIG-I [6], Cardif [8-10], TRIF [7,12], IKKε/TBK1 [13,14] and IRF3 are the key components in IFN gene activating pathways.

### Cardif cleavage by NS3/4A but not by NS2 inhibits RIG-I and Cardif-induced IFN-β promoter activity

**Figure 4:**
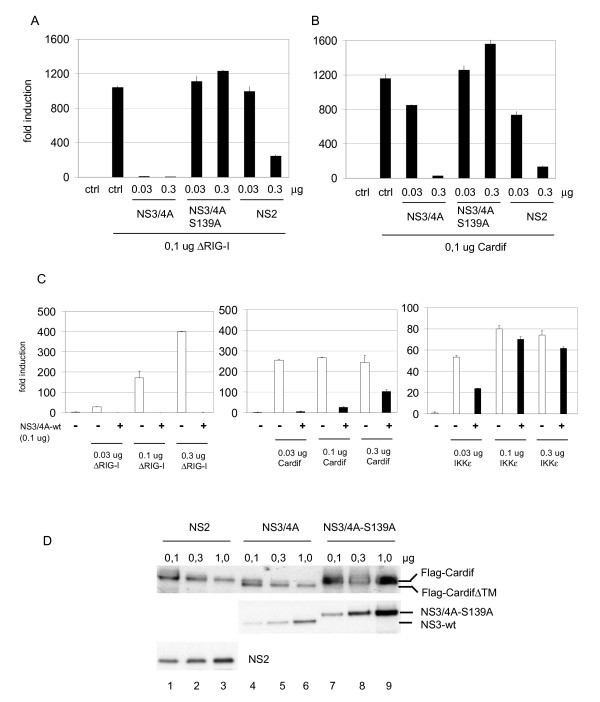
**HCV NS2 and NS3/4A inhibit RIG-I and Cardif-induced IFN promoter activity**. HEK293 cells were transfected with ΔRIG-I (constitutively active form of RIG-I) (A) or Cardif (B) expression plasmids alone or together with NS3/4A, NS3/4A-S139A or NS2 expression constructs (0.03 μg or 0.3 μg). IFN-β promoter activities were measured in cell lysates as described in the legend for Figure 1. Relative IFN-β promoter activities standardized with *Renilla *expression. (C) IFN-β promoter was induced by transfecting with increasing (0.03–0.3 μg) amounts of ΔRIG-I, Cardif or IKKε expression constructs either alone or together with NS3/4A (0.1 μg) expression construct. The effect of NS3/4A on IFN-β promoter activities were measured in HEK293 cell lysates as described in the legend for Figure 1. (D) Cells were transfected with Cardif and increasing amounts (0.1–1.0 μg) of NS2, NS3/4A and NS3/4A-S139A expression constructs. Total cell lysates were prepared and Cardif and viral protein expression was visualized by western blotting.

Since we were able to reconstitute IFN-β gene expression in HEK293 cells by overexpressing different components of the RIG-I pathway we studied whether NS2 and NS3/4A would interfere with RIG-I and Cardif-induced IFN-β promoter activity. Cells were transfected with ΔRIG-I (Fig. 4A) or Cardif (Fig. 4B) expression plasmids alone or together with NS3/4A, NS3/4A-S139A (a protease-inactive mutant of NS3/4A) or NS2 expression constructs. NS3/4A and NS2 inhibited both ΔRIG-I and Cardif-induced IFN-β promoter activity. ΔRIG-I-induced promoter activity was abolished by low amounts (0.03 μg) of NS3/4A expression plasmids (Fig. 4A) while higher amount (0.3 μg) of NS3/4A plasmid was needed to downregulate Cardif-induced activity (Fig. 4B). Protease-inactive mutant NS3/4A-S139A did not inhibit the IFN-β promoter demonstrating that the protease activity is a prerequisite for the action of HCV NS3/4A. Interestingly, lower expression levels (0.03 and 0.3 μg of plasmid vs. 1 μg used in Fig. 1) of NS2 protein specifically inhibited both ΔRIG-I and Cardif-induced IFN-β promoter activities as well (Fig. 4A and 4B). This suggests that, in addition to NS3/4A, NS2 is a potent inhibitor of cytokine gene expression.

The roles of RIG-I, Cardif and IKKε were studied when cells were transfected with increasing amounts of ΔRIG-I, Cardif or IKKε expression plasmids alone (Fig. 4C, white columns) or together with NS3/4A expression construct (Fig. 4C, black columns). NS3/4A was shown to abolish ΔRIG-I and Cardif-induced IFN-β promoter activity. The promoter activity was weakly restored with higher amounts of Cardif expression plasmid (from 0.03 ug to 0.3 μg) indicating that Cardif is partially able to overcome the inhibitory effect of NS3/4A. IKKε-induced activity was not inhibited by NS3/4A suggesting that IKKε is able to overcome the NS3/4A-mediated inhibition of IFN-β promoter (Fig. 4C). All together, the data suggest that HCV NS3/4A is likely to act only upstream from IKKε, and Cardif is rate limiting in this experimental setting.

Cardif has been shown to be a proteolytic target for HCV NS3/4A [11,20]. We studied whether NS2 utilizes a similar mechanism to inhibit IFN gene expression. Cells were transfected with Flag-Cardif and increasing amounts of HCV NS2, NS3/4A and NS3/4A-S139A expression constructs (Fig. 4D). Cardif degradation was visualized by the appearance of CardifΔTM, which is approx. 5-kDa smaller that the full-length Cardif (Fig. 4D, lanes 4–6). Higher expression of NS3/4A completely destroyed full length Cardif. Protease-inactive mutant of NS3/4A did not result in Cardif degradation indicating that the protease activity is crucial for the cleavage of Cardif by NS3/4A (Fig. 4D, lanes 6–8). NS2 protein did not degrade Cardif suggesting that inhibition of promoter activity occurs by another mechanism apart from Cardif cleavage (Fig. 4D, lanes 1–3). Together, these data indicate that NS3/4A and NS2 have different mechanisms to inhibit host cell cytokine gene expression.

### IFN/TNF-α pretreatment does not rescue cells from NS3/4A-mediated IFN-β promoter inhibition

**Figure 5:**
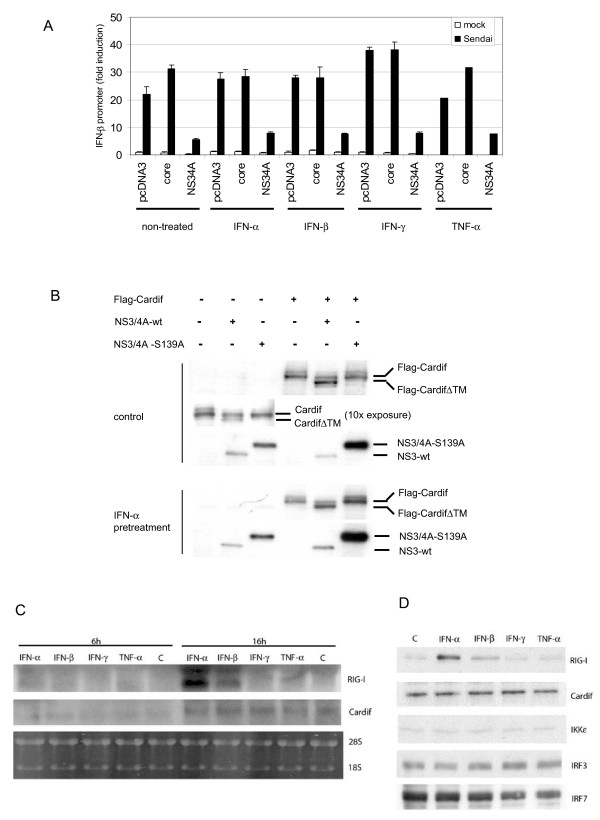
**Cytokine priming does not protect cells from HCV NS3/4A-mediated inhibition of cytokine gene expression**. (A) The effect of cytokine pre-treatment was studied in Luc-driven assay. The cells were left unprimed (non-treated) or primed with IFN-α, IFN-β, IFN-γ, (1000 IU/ml each) or TNF-α (20 ng/ml) for 18 h followed by transfection (6 h) with IFN-β promoter/*Renilla *luciferase reporter and HCV core or NS3/4A expression constructs. Transfected cells were infected with Sendai virus (MOI 5) for 16 h, cells were collected and luciferase activity was measured as indicated in the figure. The luciferase activity of the control sample was assigned to 1. (B) HEK293 cells were primed with IFN-α (1000 IU/ml) or left untreated for 16 h followed by transfection with Cardif and NS3/4A-wt or NS3/4A-S139A expression plasmids. Total cell lysate was prepared and Cardif and NS3/4A protein expression was analysed in cell lysates by immunoblotting. (C) RIG-I and Cardif mRNA was analysed in cytokine stimulated HEK293 cells. HEK293 cells were untreated (c) or stimulated with IFN-α, IFN-β, IFN-γ (1000 IU/ml each) or TNF-α (20 ng/ml) for 6 h or 16 h. Total cellular RNA was isolated and RNA samples (10 μg/lane) were analysed by Northern blotting with RIG-I and Cardif-specific cDNA probes. (D) HEK293 cells were untreated (c) or stimulated as above with IFN-α, IFN-β, IFN-γ or TNF-α for 24 h. Total cell lysate was prepared and RIG-I, Cardif, IKKε, IRF3 and IRF7 protein expression was detected by immunoblotting.

Certain cytokines may mediate strong positive feedback regulation that enhances virus-induced IFN gene expression. In many different cell types such as macrophages, dendritic cells and epithelial cells IFN-α stimulation leads to upregulation of TLR genes, TLR-associated adaptor molecules, components of the RIG-I pathway as well as IRF7 [4,24-27]. In addition to IFN-α, TNF-α pretreatment was shown to strongly enhance chemokine and IFN gene expression in influenza virus-infected lung epithelial cells as compared to non-pretreated cells [28]. Based on these findings, we studied whether IFN or TNF-α priming can overcome the inhibitory functions of NS3/4A and rescue Sendai virus-induced IFN-β gene expression (Fig. 5). It was found out that cytokine pretreatments did not have any effect on IFN-β promoter activity in HCV core or NS3/4A-expressing cells (Fig. 5A).

We also studied whether IFN-α pretreatment affects NS3/4A proteolytic activity and its capacity to degrade Cardif. Immunoblotting analysis of the cell lysates showed Cardif to be ca. 80 kDa in size (Fig. 5B). Coexpression of NS3/4A-wt, but not that of a proteolytically inactive form of NS3/4A-S139A, resulted in a faster migrating form of Cardif (approx. 5-kDa smaller) suggesting that Cardif was proteolytically cleaved by enzymatically active NS3 protein. Longer exposure (10×) of the film showed that endogenous Cardif was also sensitive to NS3/4A cleavage. IFN-α priming did not protect Cardif from NS3/4A-mediated proteolysis.

In primary human leukocytes and lung epithelial cells IFN-α or TNF-α priming enhance the expression of the components of the RIG-I pathway [24,26]. Therefore, we analyzed whether also in HEK293 cells the expression of RIG-I and/or its downstream components are induced by IFNs or TNF-α. Northern blot analysis revealed that IFN-α and to a lesser extent IFN-β induced RIG-I mRNA expression, while Cardif expression remained virtually unchanged (Fig. 5C). Western blot analysis showed that RIG-I protein expression was induced by IFN-α/β, while neither IFNs nor TNF-α was able to enhance Cardif, IKKε, IRF3 or IRF7 protein production (Fig. 5D). However, enhanced RIG-I expression was not able to overcome NS3/4A-mediated inhibition of IFN-β gene expression. This is most likely due to the fact that the expression of Cardif, the proteolytic target of NS3/4A protein complex, is not enhanced by cytokine stimulation and it thus functions as the "bottleneck" in RIG-I activated signalling pathway. Therefore, the data demonstrate that unlike in many viral infections, cytokine priming does not protect cells from HCV NS3/4A-mediated inhibition of cytokine gene expression.

### HCV NS3/4A colocalizes with Cardif at mitochondrial membrane

**Figure 6:**
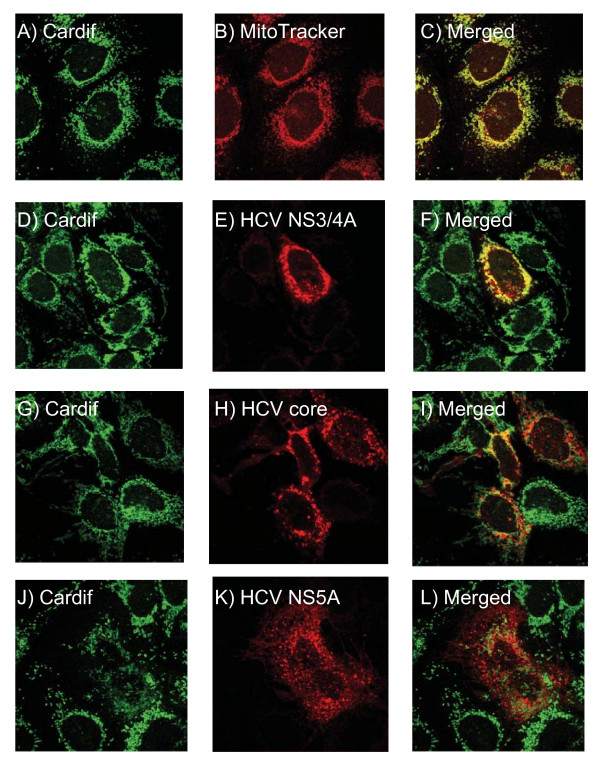
**HCV NS3/4A colocalizes with Cardif at mitochondrial membrane**. The localization of HCV proteins and Cardif was studied in Huh7 cells. The cells were transfected with HCV protein expression constructs (NS3/4A, core or NS5A) and 48 h later cells were fixed and stained. The colocalization was visualised by confocal microscopy. Cells were stained for Cardif (endogenous) (A, D, G, J), mitochondria with Mitotracker Red 580 (B), NS3/4A (E), core (H) and NS5A (K) and the signals were merged (C, F, I, L).

Recent reports have shown that Cardif localizes to the outer mitochondrial membrane, where it is the target for NS3/4A proteolysis [9,11,20]. HCV NS3/4A was shown to localize into ER and/or mitochondrion-associated membrane structures [20,29]. We studied whether NS3/4A or some other HCV proteins colocalized with endogenous Cardif, since overexpressed proteins are often mislocalized in cells. Cardif showed an excellent colocalization with MitoTracker indicating a strong mitochondrial association of Cardif in Huh7 cells (Fig. 6A–C). NS3/4A staining showed both a punctate pattern in the cytosol of the cells and significant colocalization with Cardif (Fig. 6D–F). The data is in line with another recent report [20]. It is of interest that also HCV core protein showed partial but significant colocalization with Cardif (Fig. 6G–I). Previously, core protein was demonstrated to form a granular staining pattern in the cytoplasm and associate with lipid storage vesicles and ER that may have vacuolar transport to mitochondria as well [30-32]. NS5A protein, instead, did not show any colocalization with Cardif or the mitochondria (Fig. 6j–l). Previously, NS5A protein was shown to be an ER membrane-associated protein [33].

## Discussion

Most pathogenic viruses manipulate cellular signalling pathways for their own advantage. Several HCV proteins interfere with important host signalling events and regulate e.g. cell proliferation and apoptosis. HCV uses several different strategies to evade the antiviral response. HCV NS3/4A inhibits IFN synthesis; core interferes with IFN signalling; and core, E2 and NS5A inhibit the development an antiviral response by inhibiting the functions of host antiviral proteins [see for review [34]].

HCV serine protease NS3/4A has received special attention because of its capacity to inhibit IFN production. The inhibitory mechanism began to clarify when NS3/4A was shown to inhibit Sendai virus-induced IRF-3 activation [3]. NS3/4A blocked IRF-3 phosphorylation and recent studies demonstrated that NS3/4A can directly interfere with TLR3 and RIG-I signalling pathways by cleaving the crucial adaptor molecules, TRIF and Cardif, respectively, thus rendering these pathways inactive [11,19,20]. Our study was initiated in order to systematically investigate the potential capacity of all different HCV proteins to interfere with IFN or other cytokine/chemokine gene expression. Interestingly, NS2 and NS3/4A inhibited and NS4B enhanced IFN-β promoter activity. NS3/4A was, however, demonstrated to be a more specific inhibitor for the IFN-β promoter. When expressed in high levels NS2 and NS4B proteins regulated the control promoter activity as well. Previously, NS2 had been found to inhibit several cellular (e.g., TNF-α) and viral (e.g., CMV) promoters [35]. Gene regulatory functions for NS4B have not been previously described. The mechanism how NS2 and NS4B regulate promoter activity is presently uncharacterized. Both NS2 and NS4B are ER membrane proteins with multiple transmembrane domains [36,37]. NS2 is a short-lived protein and degraded in a phosphorylation-dependent manner [38]. Fast turnover of NS2 may be advantageous for its functions in the inhibition of gene regulation and apoptosis [39]. NS4B has been implicated in the formation of ER-derived membranous webs that is the site for HCV RNA replication [40]. The gene regulatory activity of NS2 and NS4B is an interesting addition to the growing list of their multiple functions.

NS3/4A suppressed not only IFN-β promoter but also other IFN (IFN-α1, IFN-λ1) and chemokine gene promoters (CCL5, CXCL8 and CXCL10). The inhibitory effect was detected at the mRNA and protein expression level as well (M. Sillanpää, unpublished observations). These data suggest that HCV infection has broad-spectrum inhibitory effects on host cell cytokine production. The disruption of IFN production is likely to block IFN amplification loop leading to reduced expression of both IFN genes as well as IFN-stimulated genes (ISGs) (e.g., MHC molecules). Inhibition of cytokine/chemokine and ISG expression in HCV infection may lead to inefficient activation of adaptive immune response and systemic immune defects [34].

Cytokine production pathway is triggered by viral dsRNA which is produced during RNA virus replication. RIG-I and melanoma differentiation associated gene-5 (MDA-5)-stimulated pathway was recognized as a TLR3-independent dsRNA-activated signalling pathway [5,6]. It seems that TLR3 and RIG-I-induced signaling pathways are not redundant and they are often operative in different cell types [41]. Recently, Cardif/IPS-1/MAVS/VISA was identified to be RIG-I-associated adaptor molecule activating IKKα/β/γ complex, IKKε and TBK-1 leading to IRF3 phosporylation and IFN gene expression [8-11]. RIG-I and Cardif-induced IFN promoter activity was clearly inhibited by NS3/4A. The inhibitory effect was dependent on protease activity of NS3/4A and Cardif cleavage, since protease-dead NS3/4A-S139A was not able to inhibit IFN (IFN-α/β/λ) promoters and degrade Cardif. Notably, the inhibitory effect of NS2 was not mediated by Cardif cleavage. Overexpression of IKKε restored IFN-β promoter activity indicating that NS3/4A-mediated block was upstream from IKKε. In addition, overexpression of Cardif may also partially overcome the NS3/4A-mediated inhibitory effects on virus-induced IFN gene activation. These data are in line with reports showing that Cardif is the proteolytic target for NS3/4A [11,20]. The proteolysis is likely to occur at the mitochondrial membrane where Cardif and NS3/4A are colocalized. In the presence of NS3/4A, the majority of Cardif became cytosolic suggesting proteolytic cleavage and release from the mitochondrial membrane [20]. Further studies are warranted to clarify the role of mitochondria in antiviral signalling.

Cytokine production is suppressed by many viruses such as influenza A virus. IFN-α or TNF-α pre-treatment prior to virus infection may restore cell machinery to induce the IFN production. Influenza A virus infection results in a weak cytokine response while pre-treatment prior to virus infection dramatically enhanced host cell cytokine and chemokine production [25,26,28]. IFN-α or TNF-α treatment has been shown to enhance the expression of the components of the TLR3 and RIG-I pathways in human lung epithelial cells [24,28]. Enhanced expression of RIG-I and IKKε promote dsRNA recognition and IRF3 phosphorylation, respectively [6,13,14]. In the present study NS3/4A-suppressed IFN promoter activity was, however, not restored by IFN or TNF-α pre-treatment. RIG-I expression was enhanced by the stimulation of the cells with IFN-α/β, while the expression of Cardif or its downstream components were not induced. Thus, even a dramatic RIG-I expression was not sufficient to rescue the NS3/4A-mediated block in the pathway possibly due to the fact that Cardif expression was not enhanced, and it functions as the bottleneck in the RIG-I pathway.

## Conclusion

The present study exhibits systematic analysis of all HCV proteins in regulating IFN-β promoter. Serine protease NS3/4A is a crucial viral component in regulating the activation of innate immune responses. However, other viral proteins, specifically NS2 and NS4B, have a potential to regulate cytokine gene expression as well. More detailed knowledge of these proteins would help in the development of even new antivirals that would target these components of the virus. Although the mechanisms how NS3/4A is suppressing cytokine response have become at least in major part uncovered, the role of the other HCV proteins in manipulating host immune responses are still relatively poorly understood. This is, undoubtedly, an important task for the future studies.

## Methods

### Cell culture and viruses

HEK293 cells (ATCC CLR1573) and human hepatocellular carcinoma cells (Huh7) cells were cultured in Eagle's MEM supplemented with 0.6 ug/ml penicillin, 60 ug/ml streptomycin, 2 mM L-glutamine and 10% heat-inactivated FCS. Sendai virus (strain Cantell) was grown in 11-day-old embryonated chicken eggs as described [42].

### Cytokines

Purified human leukocyte IFN-α and IFN-γ were kindly provided by Dr. H. Tölö from the Finnish Red Cross Blood Transfusion Service. IFN-β was purchased from Schering-Plough and TNF-α from R&D Biosystems.

### SDS-PAGE and immunoblotting

For western blot, samples were prepared as described [25]. The blots were stained with rabbit anti-RIG-I (dilution 1:1000) [28], guinea pig anti-Cardif (1:1000), rabbit anti-IKKε (1:250) [26], rabbit anti-IRF3 (1:200) (Santa Cruz Biotechnology), rabbit anti-IRF7 (1:1000) [26], mouse anti-HCV 1a NS2 (gift from Prof. C. Rice) or mouse anti-HCV NS3 (1:400) (US Biological) antibodies in PBS containing 0,05% Tween and 1% skimmed milk powder. Anti-Cardif antibodies were prepared in guinea pigs by immunizing the animals for 4 times (50 μg/immunization/animal) at 4 week intervals with *E. coli*-expressed GST-Cardif-C (amino acids 157–540) antigen. Peroxidase-conjugated anti-mouse, anti-guinea pig and anti-rabbit secondary antibodies were from DAKO.

### RNA isolation and Northern blot analysis

Total cellular RNA was isolated by Rneasy kit (Qiagen). RNA samples (10 μg/lane) were size-fractionated on 1% formaldehyde-agarose gels and transferred to nylon membranes (Hybond:Amersham). Membranes were hybridised with probes for RIG-I and Cardif [26]. The probes were labelled with [α-^32^P] dATP using a random primed DNA labelling kit (Boehringer Mannheim).

### DNA constructs

NS3/4A and F protein genes were amplified by PCR and inserted into the BamHI site of pcDNA3.1(+)-FLAG-tagged expression vector [43]. Primers for NS3/4A; 5'-AAGGGGGGATCCACCATGGCGCCCATCACGGCGTACGCCCAGCAG-3', 5'-GTACGGGGATCCTTATCAGCACTCTTCCATCTCATCGAACTCCTG-3', F gene; 5'-AAAAAAAAGGATCCACCATGGCACGAATCCTAAACCTCAAAGA-3', 5'-TTTCCCTGGGATCCTTATCACGCCGTCTTCCAGAACCCG-3' (initiation codon underlined). Preparation of other HCV protein expression constructs have been described elsewhere [32]. The mutant NS3/4A-S139A was created using a site-directed mutagenesis kit (Stratagene). Expression plasmids for TRIF, RIP1, PI3K and Akt were kind gifts from Drs. K. Fitzgerald, G. Barber and G. Sen, respectively. Expression constructs for RIG-I, ΔRIG-I [6], IKKε, TBK1 [14], IRF3 [44] are described elsewhere. The cDNA encoding Cardif was amplified from a T cell cDNA library and cloned into pcDNA3.1(+)-FLAG. The promoter-reporter constructs of pRANTES-Luc, pIFN-β-Luc and pIFN-α4-Luc were described previously [45,46]. Luc reporter constructs under CXCL10/IP-10, CXCL8/IL-8, and TNF-α promoters were provided by Drs. R. Ransohoff, M. Kracht and J. Economou, respectively. The pIFN-λ1/IL-29-Luc, pIFN-λ3/IL-28B-Luc and pIFN-α1-Luc promoter-reporter constructs have been created as follows. The luciferase gene with SV40 mRNA polyadenylation signal was cloned into plasmid pcDEF3, resulting in plasmid pEF-Luc. Promoter fragments of the human IFN-λ1, IFN-λ3 and IFN-α1 genes were amplified with primers 5'-GGGACGCGTTTAAACCAATGGCAGAAGCTCC-3' and 5'-TGCGGTACCGGCTAAATCGCAACTGCTTCCCCAG-3' (for IFN-λ1 promoter), 5'-GCAACGCGTCATATTCCTGAGTCCTTCCTTGC-3' and 5'-CCCGGTACCGTCTGTGTCACAGAGAGAAAGGGAG-3' (for IFN-λ3 promoter), 5'-ATGACGCGTGAAATTCAGGAGTAATCAGATC-3' and 5'-GAGGTACCCGTAGATATTGCAGATACTTCTG-3' (for IFN-α1 promoter) cloned into plasmid pEF-Luc.

### Transfection and luciferase reporter assay

HEK293 cells were grown on 24-well culture plates and transfected by using FuGene6 transfection reagent (Roche Molecular Biochemicals). Cells were transfected with indicated expression plasmids together with 0.1 μg firefly luciferase reporter plasmids and 0.05 μg pRL-SV40 (*Renilla *luciferase) plasmids (Promega). At 18 h after transfection the cells were mock infected or infected with Sendai virus (MOI 5) for 24 h. The luciferase activities were determined using the Dual-Luciferase Reporter Assay System (Promega) and Victor multilabel reader (Wallac).

### Cardif cleavage *in vivo*

HEK293 cells were grown on 6-well plates and treated with IFN-α (1000 IU/ml) for 16 h. Cells were transfected with 0.25 μg of Cardif and 1 μg of NS2, NS3/4A-wt or NS3/4A-S139A expression plasmids followed by 24 h incubation. Preparation of total cell lysate and immunoblotting were carried out as described above.

### Immunofluorescence staining

Huh7 cells were transfected with pcDNA3.1(+)-FLAG-NS3/4A/core/NS5A plasmids for 48 h. The cells were fixed with 3% paraformaldehyde in PBS for 15 min, permeabilized with 0,1% Triton X-100 in PBS for 5 min. Staining of mitochondria was done in 200 nM Mitotracker Red 580 (Molecular probes) for 30 min at RT. Antibody stainings were carried out at +37°C in 0,5% BSA/PBS for 1 h. Double-stainings were done first with guinea-pig antibody against Cardif (dilution 1:100) and then with mouse HCV NS3 antibody (US Biological) (dilution 1:40) or with rabbit antibodies against HCV core (dilution 1:50) or HCV NS5A (dilution 1:200) [32]. The samples were treated with IgG-FITC or IgG-Rhodamine RedX conjugate secondary antibodies (dilution 1:100). The samples were examined using a Leica TCS NT confocal microscope with an 100× oil immersion lens. The acquired FITC and Rhodamine RedX image pairs were automatically merged with appropriate program.

## Competing interests

The author(s) declare that they have no competing interests.

## Authors' contributions

PK participated in the design of the study, performed most of the experiments, analysed the results and drafted the manuscript. MS participated in the design of the study and carried out some experiments. SK, RL and JH provided crucial reagents to carry out the experiments and helped to draft the manuscript. KM participated in the design of the study and helped with the confocal microscopy. IJ initiated the study, participated in its design and coordination and helped to draft the manuscript. All authors have read and approved the final version of the manuscript.
